# Is an X-ray a Useful Test for Esophageal Food Boluses? A Case Report

**DOI:** 10.21980/J8Q639

**Published:** 2020-07-15

**Authors:** Meryl M Abrams, Jennifer L White, Jeffrey Gardecki

**Affiliations:** * Thomas Jefferson University Hospitals, Department of Emergency Medicine, Philadelphia, PA

## Abstract

This is a case supporting the use of plain film radiography in a patient presenting with symptoms suggestive of an esophageal food bolus impaction. The patient presented with a foreign body sensation in her chest after eating chicken the night before. She complained of nausea after oral intake. She had an emergency department (ED) visit with a plain film of the chest and chest pain work up the night prior to presentation. On her return visit to the ED, the patient appeared to tolerate oral intake, but a plain film demonstrated a fluid level in the esophagus with proximal dilation. In light of this plain film finding, the patient underwent emergent endoscopy with prompt removal of the food bolus and diagnosis of the underlying etiology. This case report suggests that if a patient is able to tolerate a small volume of oral liquids yet remains symptomatic, a plain film of the chest may be helpful in diagnosis of an obstruction with a radiolucent food bolus.

**Topics:**

Plain film, esophageal food bolus impaction, esophagitis, esophageal dysmotility, obstruction.

**Figure f1-jetem-5-3-v4:**
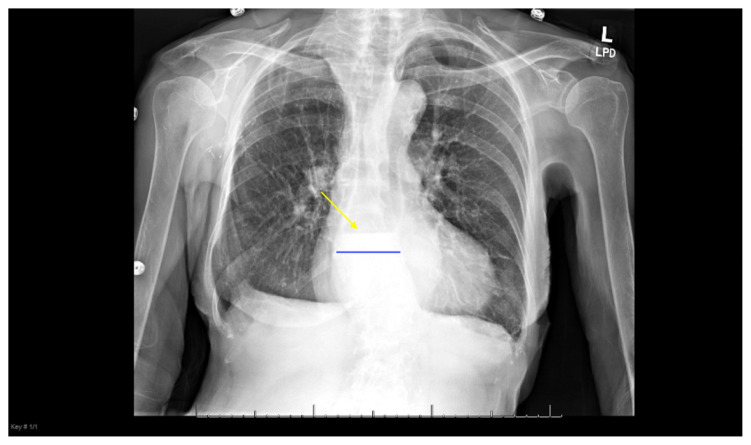


## Introduction

[Fig f1-jetem-5-3-v4]Esophageal impaction secondary to a food bolus is common and fortunately often resolves spontaneously. 1 Meat represents the most common food source responsible for esophageal obstructions in adults.^1^,^2^ The definitive treatment for this condition is endoscopic removal. There are many anecdotal treatments, such as using a carbonated beverage, glucagon, nitroglycerin or meat tenderizer. However, the only treatment with over a 50% success rate is endoscopic removal.^2^ It is key for endoscopy to be performed within 24 hours of food impaction to prevent complications. 3 A potentially life-threatening complication includes esophageal perforation and subsequent mediastinitis. Therefore, accurate diagnosis of an esophageal food bolus impaction is imperative in order to avoid delay of treatment. This case report suggests that a plain film radiograph of the chest after oral intake of fluids may facilitate prompt diagnosis of an esophageal food impaction.

## Presenting concerns and clinical findings

An 89-year-old female with a past medical history significant for coronary artery disease and hyperlipidemia presented to the ED with the sensation of food being stuck in her mid-chest. The patient had chicken soup the night prior to presentation and had been unable to tolerate oral intake since that time. She came to the emergency department the night prior and had an unchanged electrocardiogram, a normal plain film of the chest and negative serial troponins. She tolerated minimal oral intake and was discharged with return precautions. She returned to the emergency department because she was unable to tolerate her morning medications due to nausea and vomiting. Her vital signs were: blood pressure 165/79 mmHg, temperature 97.7°F, heart rate 69 beats per minute, respiratory rate 20 breaths per minute and 97% oxygen saturation on room air. She had a normal cardiac examination, clear lung sounds and a soft, nontender, non-distended abdomen. A repeat electrocardiogram was unchanged from prior. She was given a cup of water, which she drank without difficulty. She noted some nausea but did not vomit. A chest x-ray was taken.

## Significant findings

The plain film radiograph of the chest demonstrated a fluid level (yellow arrow) in the distal esophagus with dilation of the esophagus proximal to that point (blue line). Neither of these findings were present on the previous visit.

## Patient course

Gastroenterology was consulted and agreed to take the patient for endoscopy. The endoscopy demonstrated food particles in the distal esophagus, esophagitis and esophageal dysmotility. There were no complications from the procedure. She was admitted overnight for observation given her cardiac risk factors and age. She was discharged the following day on a proton pump inhibitor with gastroenterology follow up for further investigation of her esophageal dysmotility and esophagitis. A barium swallow study was ordered but has not been performed yet. Telehealth visits have demonstrated improvement of the patient’s symptoms and adherence to her medications.

## Discussion

The strength of this case report is that it demonstrates that a plain film of the chest may be helpful in diagnosis of an esophageal food bolus impaction and allow for prompt definitive treatment. X-rays are typically used to assess for radiopaque foreign bodies or perforation. Prior studies have shown food bolus impactions on chest computed tomography (CT) and barium swallow, but the investigators were unable to identify prior reports of non-contrast plain films revealing esophageal fluid levels secondary to food bolus impaction. 2 Plain films deliver less radiation than CT and do not have the risk of aspiration of contrast associated with barium swallow studies. There are minimal data on the sensitivity and specificity of plain films to detect esophageal food boluses; however, prior studies have shown that plain films may have a false negative rate of 47% and a sensitivity of around 30%.^4^ The rapidity with which plain films can be performed may also lead to more prompt definitive treatment and fewer potential complications.

The treatment of food bolus impactions can be with expectant management, medications, or endoscopic removal. Expectant management, however, carries the risk of complications secondary to a missed esophageal micro perforation and subsequent mediastinitis. It is important to be prepared for airway management should the patient be unable to tolerate the secretions due to the esophageal obstruction. Medical management may include intravenous glucagon; however, its efficacy in symptom resolution is only a little greater than 50%.^5^ Endoscopy has been shown to be almost 100% effective in the treatment of food bolus impactions.^6^

The main weakness of this case report is that it is an isolated case, and additional research needs to be done in order to demonstrate a statistically significant difference in outcomes with early plain films. The other limitation is that for the plain film to be useful in the manner suggested, the patient must be able to tolerate a small amount of oral intake. The sensitivity and specificity of plain films for esophageal food bolus impaction is unknown since it is currently not common practice unless the provider suspects perforation or bony food bolus.^6^ This case report demonstrates an additional utility of plain film radiography of the chest in a patient with suspected esophageal food bolus impaction.

## Supplementary Information





## References

[1] Long B Koyfman A Gottlieb M Esophageal foreign bodies and obstruction in the emergency department setting: an evidence-based review J Emerg Med 2019 56 499 511 10.1016/j.jemermed.2019.01.025 30910368

[2] Elango S Palaniapan SP Lingam VS George L Esophageal food bolus impaction Singapore Medical Journal 1990 31 624 626 2281364

[3] Ikenberry SO Jue TL Anderson MA Management of ingested foreign bodies and food impactions GIE 2011 73 6 1085 1091 10.1016/j.gie.2010.11.01 21628009

[4] Chirica M Kelly MD Siboni S Esophageal emergencies: WSES guidelines World J Emerg Surg 2019 14 26 10.1186/s13017-019-0245-2 31164915 PMC6544956

[5] Glauser J Lilja GP Greenfeld B Ruiz E Intravenous glucagon in the management of esophageal food obstruction JACEP 1979 8 6 228 231 10.1016/s0361-1124(79)80184-7 449146

[6] Ko HH Enns R Review of food bolus management Can J Gastroenterol 2008 22 10 805 808 10.1155/2008/682082 18925301 PMC2661297

[7] Loh KS Tan LK Smith JD Yeoh KH Dong F Complications of foreign bodies in the esophagus Otolaryngol Head Neck Surg 2000 123 613 616 11077351 10.1067/mhn.2000.110616

[8] O’Donnell C Esophageal bolus obstruction: Radiology Case Radiopaedia Blog RSS https://radiopaedia.org/cases/oesophageal-bolusobstruction?lang=us Accessed April 4, 2020

[9] Kragha KO Complete gastroesophageal obstruction by food bolus Appl Radiol 2016 45 9 40 44

