# Supplementary figures and images for: Is an X-ray a Useful Test for Esophageal Food Boluses? A Case Report
Source: J Educ Teach Emerg Med. 2020 Jul 15;5(3):V4–6. doi: 10.21980/J8Q639 (PMC10332547; doi:10.21980/J8Q639)

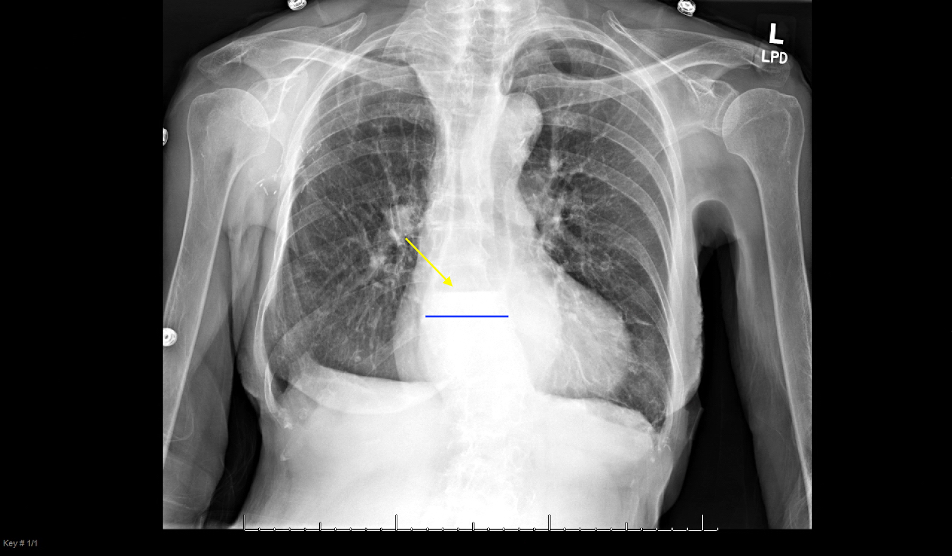

Supplement: Supplementary file 1 [file jetem-5-3-v4-supp1.jpg]

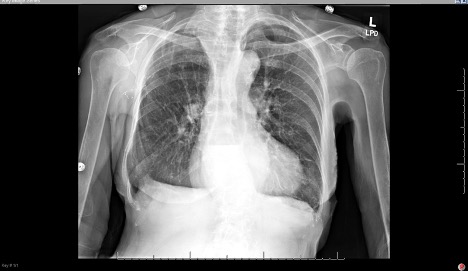

Supplement: Supplementary file 2 [file jetem-5-3-v4-supp2.jpg]
